# Mitochondrial-targeted antioxidant supplementation for improving age-related vascular dysfunction in humans: A study protocol

**DOI:** 10.3389/fphys.2022.980783

**Published:** 2022-09-15

**Authors:** Kevin O. Murray, Morgan Berryman-Maciel, Sanna Darvish, McKinley E. Coppock, Zhiying You, Michel Chonchol, Douglas R. Seals, Matthew J. Rossman

**Affiliations:** ^1^ Integrative Physiology of Aging Laboratory, Department of Integrative Physiology, University of Colorado Boulder, Boulder, CO, United States; ^2^ Division of Renal Diseases and Hypertension, University of Colorado Anschutz Medical Campus, Aurora, CO, United States

**Keywords:** aging, reactive oxgen species, endothelial function, arterial stiffness, mitochondria

## Abstract

**Background:** Cardiovascular disease (CVD) is the leading cause of death worldwide and aging is the primary risk factor for the development of CVD. The increased risk of CVD with aging is largely mediated by the development of vascular dysfunction. Excessive production of mitochondrial reactive oxygen species (mtROS) is a key mechanism of age-related vascular dysfunction. Therefore, establishing the efficacy of therapies to reduce mtROS to improve vascular function with aging is of high biomedical importance. Previously, in a small, randomized, crossover-design pilot clinical trial, our laboratory obtained initial evidence that chronic oral supplementation with the mitochondrial-targeted antioxidant MitoQ improves vascular function in healthy older adults. Here, we describe the protocol for an ongoing R01-funded phase IIa clinical trial to establish the efficacy of MitoQ as a therapy to improve vascular function in older adults (ClinicalTrials.gov Identifier: NCT04851288).

**Outcomes:** The primary outcome of the study is nitric oxide (NO)-mediated endothelium-dependent dilation (EDD) as assessed by brachial artery flow-mediated dilation (FMD_BA_). Secondary outcomes include mtROS-mediated suppression of EDD, aortic stiffness as measured by carotid-femoral pulse wave velocity, carotid compliance and β-stiffness index, and intima media thickness. Other outcomes include the assessment of endothelial mitochondrial health and oxidative stress in endothelial cells obtained by endovascular biopsy; the effect of altered circulating factors following MitoQ treatment on endothelial cell NO bioavailability and whole cell and mitochondrial reactive oxygen species production *ex vivo*; and circulating markers of oxidative stress, antioxidant status, and inflammation.

**Methods:** We are conducting a randomized, placebo-controlled, double-blind, parallel group, phase IIa clinical trial in 90 (45/group) healthy older men and women 60 years of age or older. Participants complete baseline testing and are then randomized to either 3 months of oral MitoQ (20 mg; once daily) or placebo supplementation. Outcome measures are assessed at the midpoint of treatment, i.e., 6 weeks, and again at the conclusion of treatment.

**Discussion:** This study is designed to establish the efficacy of chronic supplementation with the mitochondrial-targeted antioxidant MitoQ for improving vascular endothelial function and reducing large elastic artery stiffness in older adults, and to investigate the mechanisms by which MitoQ supplementation improves endothelial function.

## Introduction

### Background and rationale

**FIGURE 1:**
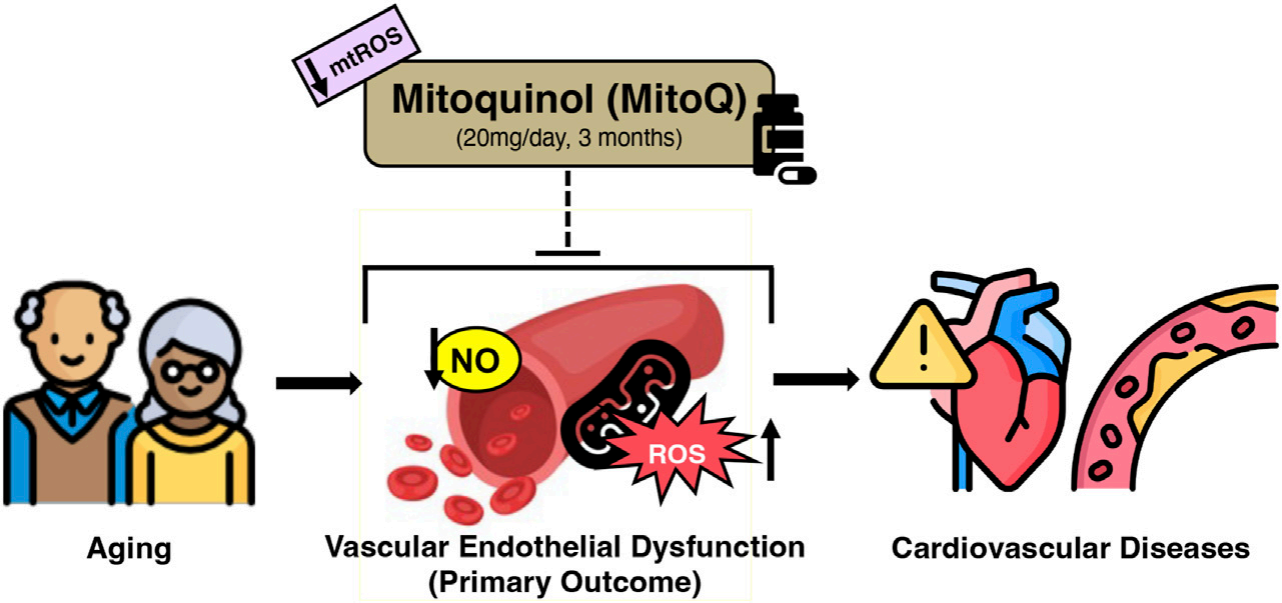
Working hypothesis of MitoQ supplementation for improving age-related declines in vascular endothelial function in healthy older adults. Created with resources from flaticon.com.

Cardiovascular disease (CVD) remains the leading cause of death in the U.S. and more than 65% of adults over 60 years of age develop CVD (Benjamin et al., 2017). Advancing age is the strongest independent risk factor for CVD and, as such, the majority of deaths from CVD occur in adults aged 60 years of age or older (Lakatta, 2003; Lloyd-Jones, 2010; Benjamin et al., 2017). The increased risk of CVD with age is mediated largely by vascular dysfunction, primarily endothelial dysfunction, as assessed by impaired nitric oxide (NO)-mediated endothelium-dependent dilation (EDD), and stiffening of the large elastic arteries (i.e., the aorta and carotid arteries) (Lakatta and Levy, 2003; Widlansky et al., 2003; Brandes et al., 2005; Mitchell et al., 2010; Seals et al., 2011). Excessive production of reactive oxygen species by mitochondria (mtROS) is a major source of vascular oxidative stress and an emerging driver of vascular dysfunction with aging (James and Murphy, 2002; Lakatta and Levy, 2003; Fleenor et al., 2010; Seo et al., 2010; Seals et al., 2011; Fleenor, 2013; López-Armada et al., 2013). Given projected increases in the number of older adults and consequent CVD prevalence (Olshansky et al., 2009; Heidenreich et al., 2011), establishing novel strategies that promote healthy vascular aging by reducing mtROS is an important biomedical research objective (Figure 1) (Cochemé et al., 2007; Lakshminarasimhan and Steegborn, 2011; Dai et al., 2012; Dromparis and Michelakis, 2013; Kluge et al., 2013).

Mitoquinol (MitoQ) is a mitochondria-targeted antioxidant consisting of the naturally occurring antioxidant ubiquinol, biochemically modified to accumulate in the mitochondria where it is optimally positioned to reduce mtROS (Murphy and Smith, 2007; Smith and Murphy, 2010; Heidenreich et al., 2011). Preclinical findings from our laboratory show that 4 weeks of oral MitoQ supplementation in drinking water completely restores vascular function in old (26–28 months) mice (Gioscia-Ryan et al., 2018; Gioscia-Ryan et al., 2014). Recently, we took the first step in translating these preclinical findings to humans by completing a randomized, placebo-controlled, crossover design pilot clinical trial of MitoQ supplementation in adults aged 60–80 years (*n* = 20) (Rossman et al., 2018). The results of this small trial suggest that short-term (6 weeks) MitoQ supplementation is well tolerated, improves endothelial function in healthy older adults with endothelial dysfunction at baseline, and is associated with a reduction in a circulating marker of oxidative stress, i.e., oxidized low-density lipoprotein (oxLDL) (Rossman et al., 2018). Moreover, similar to outcomes seen in old mice (Gioscia-Ryan et al., 2014), preliminary assessments in a subgroup of subjects in our pilot clinical trial suggest an amelioration of mtROS-mediated suppression of EDD, reduced endothelial cell oxidative stress, and improved vascular mitochondrial health as key mechanisms underlying the favorable effects of MitoQ on endothelial function (Gioscia-Ryan et al., 2014; Rossman et al., 2018). Moreover, MitoQ supplementation reduced aortic stiffness (carotid-femoral PWV; cfPWV) in older adults with age-related increases in aortic stiffness assessed under placebo conditions (Gioscia-Ryan et al., 2018; Rossman et al., 2018).

To extend the initial observations in our pilot trial, we are conducting a larger randomized, placebo-controlled, double-blind, single-site, parallel group design phase IIa clinical trial over a longer, more clinically relevant supplementation period (Whelton et al., 2017; Grundy et al., 2018) (3 months; 20 mg/day) to establish the efficacy of MitoQ supplementation for improving vascular function and determine the mechanisms by which MitoQ mediates the improvements in healthy older adults. To do so, we are combining gold-standard clinical measures of vascular function with innovative translational research techniques to assess: 1) NO-mediated EDD [brachial artery flow-mediated dilation, FMD_BA_ (*Primary Outcome*)]; 2) mtROS-mediated suppression of EDD (*Secondary Outcome*); 3) the impact of changes in circulating factors on endothelial cell NO bioavailability and mitochondrial and whole-cell ROS production (*Other Outcomes*); 4) endothelial cell oxidative stress and mitochondrial fitness (*Other Outcomes*); and 5) circulating markers of oxidative stress (*Other Outcomes*). We are also determining the effects of MitoQ supplementation on large elastic artery stiffness (cfPWV*, Secondary Outcome;* and carotid artery beta-stiffness index*, Other Outcomes*)*.*

## Methods and analysis

### Study design

**FIGURE 2:**
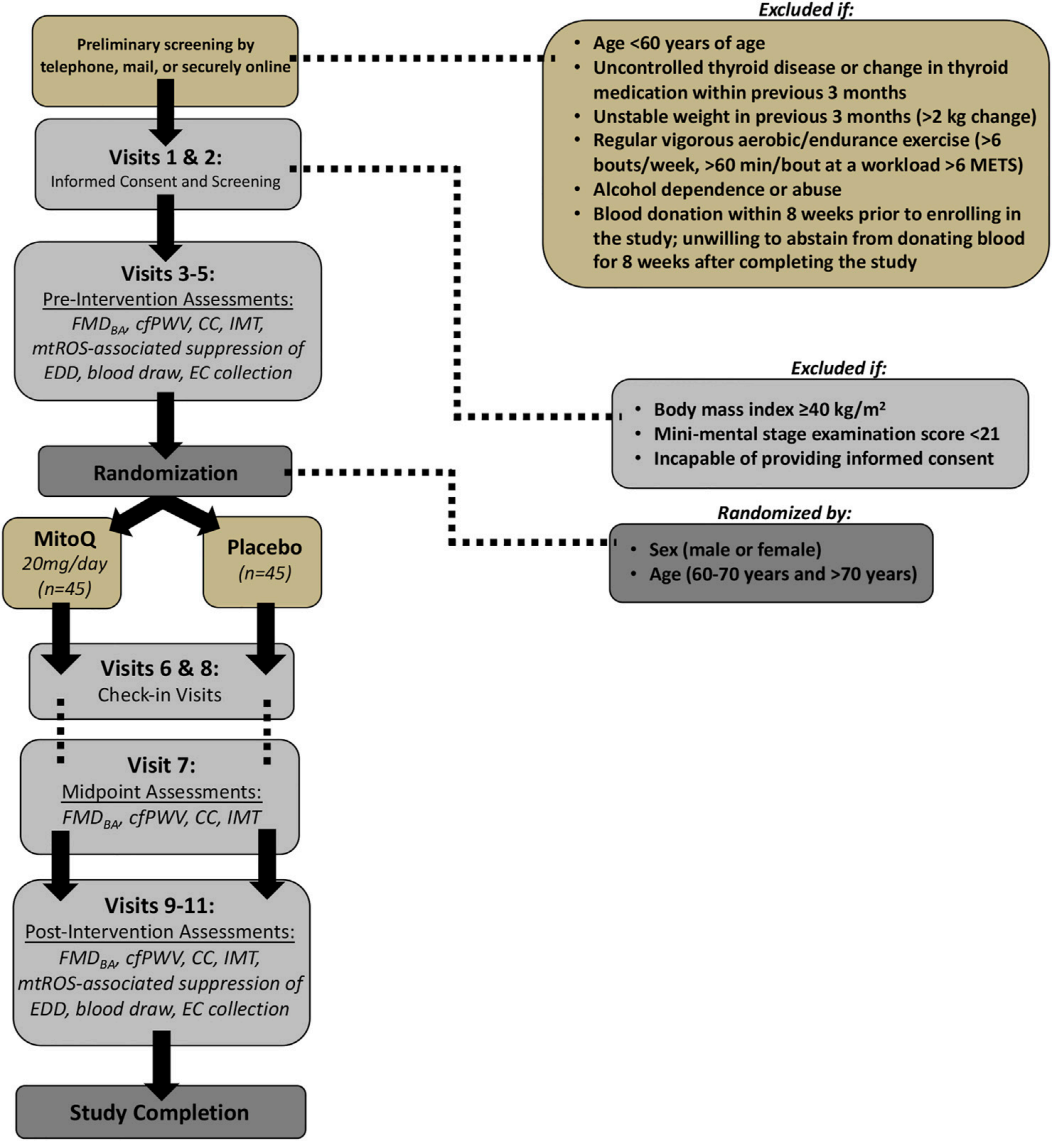
Study Design. FMD_BA_ = brachial artery flow-mediated dilation; cfPWV = carotid-femoral pulse wave velocity; CC = carotid (artery) compliance; IMT = (carotid artery) intima media thickness; EC = endothelial cell; METs = metabolic equivalents; mtROS = mitochondrial reactive oxygen species; EDD = endothelium-dependent dilation.

This is a randomized, placebo-controlled, double-blind, parallel group, phase IIa clinical trial to assess the effects of 3 months of oral MitoQ or placebo supplementation (*n* = 45/group; Figure 2) for improving vascular endothelial function (FMD_BA_; primary outcome) and the mechanisms by which MitoQ mediates those benefits in healthy older adults 60 years of age or older. We are also assessing the effect of MitoQ supplementaiton on large elastic artery stiffness (cfPWV; secondary outcome). For women, postmenopausal status is confirmed through a questionnaire; additionally, we record the time from the final menstrual period. Men and women from all ethnic backgrounds are being enrolled (Table 1).

**TABLE 1:** Inclusion and Exclusion Criteria. METs = metabolic equivalents.

Inclusion criteria	Exclusion criteria
• Age ≥60 years	• Uncontrolled thyroid disease or change in thyroid medication within previous 3 months
• Ability to provide informed consent	• Regular vigorous aerobic/endurance exercise (>6 bouts/week, >60 min/bout at a workload >6 METS)
• Willing to accept random assignment to condition	• Blood donation within 8 weeks prior to enrolling in the study; unwilling to abstain from donating blood for 8 weeks after completing the study
• Body mass index <40 kg/m2	
• Weight stable in the prior 3 months (<2 kg weight change) and willing to remain weight stable throughout the study	
• Ability to perform cognitive tests	
• Free from alcohol dependence or abuse, as defined by the American Psychiatry Association, Diagnostic and Statistical Manual of Mental Disorders (DSM-V)	
• Mini-mental stage examination score ≥21	

### Description and rationale of intervention

#### Duration and dose

MitoQ is sold as a dietary supplement with a recommended daily dose of 10 mg/day; however, there are currently no available data supporting the efficacy of this dose for improving physiological function (Shill et al., 2016). Most clinical trials with chronic MitoQ supplementation have used doses ranging from 20–80 mg/day (Gane et al., 2010; Snow et al., 2010; Rossman et al., 2018). Importantly, these studies have demonstrated that MitoQ can be administered safely to humans for up to 1 year at doses up to 80 mg/day without serious adverse events (Gane et al., 2010; Snow et al., 2010; Rossman et al., 2018).

This trial uses a dose of 20 mg/day; this is based on our pilot study showing evidence of efficacy of 20 mg/day of MitoQ for improving endothelial function (the *primary outcome* of the current study) and reducing aortic stiffness in healthy older adults with a shorter intervention duration (6 weeks) (Rossman et al., 2018). Considering this dose was also safe and well tolerated by subjects over the 6-weeks intervention (Rossman et al., 2018) (only minor gastrointestinal distress was reported) we are extending the intervention duration to a more clinically relevant period of 3 months based on the clinical guidelines for the evaluation of efficacy of CVD risk factor modifying treatments (Whelton et al., 2017; Grundy et al., 2018).

We use a single, acute 160 mg dose of MitoQ to inhibit mtROS signaling to mechanistically assess the beneficial effects of MitoQ as reported previously (Rossman et al., 2018). We have chosen this higher dose for acute administration based on its observed efficacy for acutely improving vascular function (i.e., to acutely reduce mtROS to assess its tonic inhibition of physiological function) (Park et al., 2020). This higher dose was well tolerated (no subjects dropped out of the study because of side effects) with no serious adverse events in our pilot study (Rossman et al., 2018).

#### Potential side effects

The expected side effects of MitoQ consumption are gastrointestinal distress and discomfort.

#### Compound descriptions

MitoQ and placebo tablets are provided by: MitoQ Limited, Auckland, New Zealand. MitoQ tablets are pale yellow capsules for oral administration. Each MitoQ tablet contains 20 mg of mitoquinol mesylate at a concentration of 19.4–24.4% w/w, tapioca starch, microcrystalline cellulose, hypromellose, silica-colloidal anhydrous, purified water, carrageenan, and pectin. MitoQ Limited manufactures these 20 mg tablets explicitly for clinical research studies to aid in subject adherence. Placebo tablets are identical in physical appearance to MitoQ tablets (matched in size and color) and contain a blend of microcrystalline cellulose, silicon dioxide, and tapioca.

#### Packaging & handling of compounds

Tablets are stored and dispensed by the University of Colorado (CU) Boulder Clinical Translational Research Center (CTRC) pharmacy. MitoQ and placebo pills are packaged in HDPE bottles. Per manufacturer instructions, MitoQ and placebo pills are stored between 20–25°C with desiccant, avoiding high humidity and excessive heat above 40°C. The tablets are not split, crushed, or chewed. Study participants receive a new pill supply during regular CTRC check-in visits.

#### Identity, validity, and stability of MitoQ

MitoQ or placebo pills are supplied by the manufacturer in 6 month increments such that we have an adequate amount (with minimal excess). Pills are not administered past their expiration date. Presence and stability of MitoQ in capsules is verified by certificates of analysis provided by the manufacturer and periodic LC/MS analysis performed by the CU Boulder Central Analytical Mass Spectrometry Laboratory.

#### Expected duration of intervention

The intervention involves 3 months of supplementation; however, in the event of a conflict during or after the intervention period (due to scheduling, illness, etc.), the intervention may be shortened to 2 months or lengthened to 4 months. Subjects continue their treatment (MitoQ or placebo) until all post-intervention testing has been completed. Based on the timing information above, the study takes 4 months on average to complete but may be shortened to 3 months or extended to 5 months. The study requires approximately 19 h of the subject’s time.

## Outcomes

### Primary outcome

#### Endothelial function

EDD is assessed by FMD_BA_, the gold-standard noninvasive measure of NO-mediated EDD, according to current guidelines (Thijssen et al., 2019). The brachial artery is imaged using Doppler ultrasonography (Toshiba Xario XG) (Rossman et al., 2018; Craighead et al., 2021). After an acceptable image is obtained, a blood pressure cuff placed around the forearm distal to the elbow is inflated to 250 mmHg for 5 min. Brachial artery blood flow and vessel diameter is recorded for 1 min prior to cuff inflation and for 2 min following rapid cuff deflation. EDD is determined by measuring the degree of dilation (peak change in vessel diameter from baseline diameter) and forearm reactive hyperemia is determined by measuring the blood flow response following cuff deflation (Rossman et al., 2018; Craighead et al., 2021). Shear rate is calculated as (8 x mean velocity [m/s])/occlusion diameter (m); if changes occur to shear rate, we calculate shear rate to normalize FMD_BA_ responses (Harris et al., 2010). To control for changes in smooth muscle sensitivity to NO (Seals et al., 2011), endothelium-independent dilation is determined by measuring brachial artery dilation to sublingual nitroglycerin (0.4 mg) for 10 min (Duffy et al., 2001).

### Secondary outcomes

#### mtROS-mediated suppression of endothelial function

We measure FMD_BA_ before and 1 h after an acute 160 mg oral dose of MitoQ to suppress mtROS signaling (Rossman et al., 2018). A 160 mg oral dose allows us to maximally elevate plasma concentrations of MitoQ. This acute dose was effective and well tolerated in our pilot study, with no serious adverse events (Rossman et al., 2018). The difference in FMD_BA_ after acute oral MitoQ versus baseline is taken as a measure of the tonic suppression of EDD by mtROS.

#### Large elastic artery stiffness

Large elastic artery stiffness is measured by cfPWV. Briefly, transcutaneous tonometry is used to obtain arterial pressure waveforms from the carotid and femoral arteries consecutively (Non-Invasive Hemodynamics Workstation; Cardiovascular Engineering Inc.) (Martens et al., 2018; Rossman et al., 2018). Electrocardiogram gating of the R wave is used to determine the time delay (transit time) between the foot of the pressure waves measured at each location; cfPWV is calculated as the distance between measurement sites divided by transit time of the arterial pulse wave.

Carotid artery compliance, a measure of carotid artery distensibility, is measured non-invasively using high-resolution ultrasonography (Toshiba Xario XG) and applanation tonometry (NIHem) (Tanaka et al., 2000; Seals et al., 2001) and is calculated with the following equation: {3.141592 x [(2 x carotid diastolic diameter) x (carotid systolic-diastolic diameter) + (carotid systolic-diastolic diameter)^2^]/4 x (carotid pulse pressure)} (DeVan et al., 2016). β-stiffness index is also measured and calculated with the following equation: [Ln ((carotid systolic blood pressure/carotid diastolic blood pressure))/((carotid systolic diameter - carotid diastolic diameter)/carotid diastolic diameter)]; β-stiffness index offers an additional measure of arterial stiffness that is independent of blood pressure (Hirai et al., 1989; Wada et al., 1997; DeVan et al., 2016). Arterial wall thickness is assessed by measuring intima media thickness on an ultrasound image of the carotid artery (Tanaka et al., 2002). Briefly, images of the carotid artery to assess intima media thickness are acquired within the proximal region of the straight portion of the common carotid artery for a duration of 30 s. Resulting images are measured during end diastole using a computerized image-analysis software (Medical Imaging Applications, LLC; Carotid Analyzer for Research, Version 6). Carotid intima media thickness is defined as the distance from the edge of the lumen-intima border to the edge of the media-adventitia border (Tanaka et al., 2002; Craighead et al., 2021).

### Other outcomes

#### Endothelial cell protein quantification

Two 0.025-inch in diameter J-shaped wires are placed one at a time through a venous catheter (antecubital vein) for endothelial cell collection pre- and post-intervention (Donato et al., 2007; Donato et al., 2008) (Figure 2). This technique is performed by a CTRC nurse. The endothelial cells collected by the two wires are prepared for protein expression analyses. The distal portions of the wires are transferred to a 50-ml conical tube containing a dissociation buffer that includes PBS, EDTA, heparin, and bovine serum albumin. Cells are isolated by centrifugation, fixed with 3.7% formaldehyde, and adhered to poly-l-lysine coated slides (Donato et al., 2007; Donato et al., 2008; Jablonski et al., 2011; Pierce et al., 2011). Vascular endothelial cell phenotype is confirmed by staining with vascular endothelial cadherin (VE-Cadherin) using fluorescent microscopy. The endothelial cell count per slide averages ∼55 (range 25–92, estimated total of ∼200–750 endothelial cells per subject) for samples obtained from veins. More than 90% of the endothelial cells retain nuclear integrity (determined using DAPI staining). We are quantifying cellular markers of oxidative stress (i.e., nitrotyrosine (NT) abundance) and mitochondrial health and fitness (i.e., mitochondrial fission one protein (Fis1)) to provide insight regarding the mechanisms responsible for improvements in vascular function with MitoQ supplementation.

#### Influence of circulating factors on endothelial cell NO bioavailability and reactive oxygen species production

**FIGURE 3:**
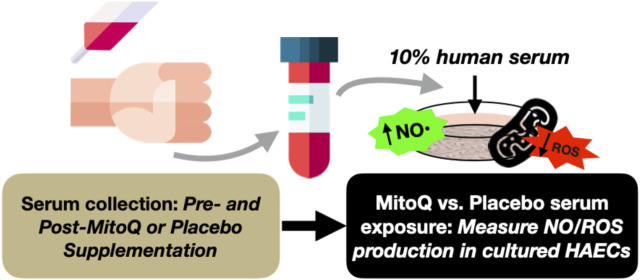
Experimental design to assess the influence of changes in circulating factors on endothelial cell health before and after MitoQ or placebo supplementation. NO = nitric oxide; ROS = reactive oxygen species; HAECs = human aortic endothelial cells.

Human aortic endothelial cells (HAECs) (PromoCell; used after 4−6 passages) are plated in 96-well culture plates and incubated under standard conditions (37 °C, 5% CO_2_, 100% humidity) for 24 h in basal media supplemented with 10% human serum collected during normally scheduled visits pre- and post-MitoQ or placebo supplementation (in triplicate) (Craighead et al., 2021)^,^ (Clayton et al., 2020). HAECs are stained with the fluorescent probes DAR-4M AM (Sigma-Aldrich; to detect NO production), CellROX Deep Red (ThermoFisher; to detect whole-cell ROS production), MitoSOX (ThermoFisher; to detect mtROS production), and MitoTracker (ThermoFisher; to control for differences in mitochondrial volume), and analyzed by quantitative fluorescence microscopy (EVOS M7000, Celleste Image Analysis Software 6.0; ThermoFisher) (Craighead et al., 2021), (Clayton et al., 2020). HAECs stained with DAR-4M are imaged before and 6 min after addition of 200 μmol/L acetylcholine (Sigma) to stimulate NO production (Figure 3).

#### Markers of oxidative stress, antioxidant status, and inflammation

Following blood draws performed during normally scheduled visits pre- and post- MitoQ or placebo supplementation (Figure 2), plasma oxLDL and myeloperoxidase, circulating markers of oxidative stress and oxidant production (Kasapoglu and Ozben, 2001; Walker et al., 2012), respectively, are determined by ELISA (Mercodia). Serum total antioxidant status (Randox Laboratories) (Kasapoglu and Ozben, 2001; Walker et al., 2012), a marker of antioxidant defenses and markers of systemic inflammation, including plasma interleukin (IL)-6 [by ELISA (Mercodia)] and C-reactive protein (CRP) [by immunoturbidimetry (Beckman Coulter)] (Donato et al., 2008) are also assessed. We are also measuring mitochondrial DNA (mtDNA) copy number (quantity), damage, and heteroplasmy (quality) at mtDNA sites associated with CVD by polymerase chain reaction in peripheral blood mononuclear cells (PBMCs) as described elsewhere (Mengel-From et al., 2014; Sobenin et al., 2015; Fetterman et al., 2016; Mitrofanov et al., 2016; Zole and Ranka, 2019).

### Selection and treatment of subjects

#### Study location and timeline

All study visits are performed on the CU Boulder campus at the campus CTRC. We anticipate needing to screen ∼230 participants for meeting inclusion/exclusion criteria *via* phone, online questionnaires, and in-person screening procedures to enroll 112 subjects over a period of 55 months. This plan accounts for an approximate 20% dropout rate of enrolled participants to ensure 90 subjects (45/group) complete the study in full (see *Statistical Analyses* section). Eligible subjects are enrolled in the trial at a rate of 2−3 subjects per month.

#### Recruitment and eligibility

Participants are recruited from Boulder County and the surrounding areas by the study coordinator and other study staff with study flyers, newspaper advertisements, recruitment emails, Facebook, Instagram, ResearchMatch.org, and Craigslist. Participants who respond to study advertising do so by email or telephone; at such time, potential participants undergo preliminary screening, which also includes pre-consent (Figure 2). Subjects choose to complete the pre-screening questions through the mail, over the phone, or online via Research Electronic Data Capture (REDCap; a secure web application designed to support data capture for research studies) depending on the participant’s preference. A study information form is also included in the materials. For privacy protection, mailed screening forms are pre-coded (to identify the participant) and participants are instructed not to write their name on the screening form.

#### Informed consent and screening

Participants who meet the inclusion criteria are then contacted and scheduled for their screening visits (*Visits 1 & 2*; Figure 2). Participants who do not meet inclusion criteria are also notified.

Our COVID-19 protocols follow the current CDC, state, local, and/or CTRC guidelines to provide the highest level of safety to the participants and staff. All COVID-19 related infections are documented throughout the study.

Informed consent is obtained in person, or electronically over Zoom, using REDCap to collect signatures (*Visit 1*; Figure 2). The study coordinator or a co-investigator explains all study procedures to potential participants prior to their participation in any experimental procedure. The study coordinator or a co-investigator asks participants questions about the study procedures to ensure participant comprehension and autonomy. If an individual decides to participate in the study, they are asked to sign the consent form in REDCap to document their consent in accordance with the Declaration of Helsinki. The study coordinator or co-investigator obtaining consent digitally signs the last page of the consent form. A copy of the signed consent form is provided to the participant. Ethical Approval for this study has been obtained from the University of Colorado Institutional Review Board (IRB) (Protocol #20-0502).

Participants who have provided informed consent undergo screening procedures, briefly listed below, performed over a 1-week period to determine if subjects qualify for the study (*Visits 1 & 2*; Figure 2). Prior to these visits, subjects are asked to abstain from non-prescribed over-the-counter medications and supplements for 48 h (>4 h allowed), food and caffeine for 5 h (>3 h allowed), alcohol and strenuous exercise for 24 h (>12 h allowed), and marijuana and tobacco for 24 h. Screening measures include:

1) Resting blood pressure and heart rate
2) Height and body weight
3) Medical history and physical examination
4) Resting electrocardiogram (ECG)
5) Questionnaires and paperwork
6) Family medical history
7) Cognitive function (Mini Mental State Exam, MMSE)
8) Physical activity (Modified Activity Questionnaire, MAQ)
9) Sleep Questionnaire
10) Blood collection to assess metabolic and lipid profiles, complete blood count, and thyroid stimulating hormone

Once screening procedures are performed, each of the potential subject’s results are reviewed by the physician of record to assess eligibility for the study.

#### Pre-intervention experimental assessments

Eligible participants who qualify for the study undergo pre-intervention experimental testing (*Visits 3–5*; Figure 2) over a 2-week period; the numerical order of visits may be rearranged to facilitate scheduling. Subjects are asked to avoid major lifestyle changes including significant changes in physical activity (monitored via accelerometer; GENEActiv; 3-days recording) and time spent doing standard weekly activities (CHAMPS questionnaire), body weight, and diet (Automated Self-Administered 24-h diet recall; ASA24; National Cancer Institute) throughout the duration of the study. Subjects also are asked to record their diet for 24 h prior to their first pre-intervention outcome measure assessment visit. Subjects are asked to adhere as closely to this diet as possible prior to each future outcome measure assessment visit, both pre- and post-intervention, to control for acute diet-related changes to outcome measures. Subjects are asked to inform the research staff if there is a change in any lifestyle habits (either physician recommended or personal choice). Subjects are asked to maintain a stable medication regimen for the duration of the study, or to notify the research team should their physician recommend a change in medication during the study. Subjects are encouraged to notify their physician about their participation in the study.

Subjects are asked to follow the same pre-visit restrictions in *Visits 3–5* (pre-intervention testing) as followed for *Visits 1 &* 2 (screening assessments). Subjects also receive their initial allotment (2 weeks) of MitoQ or placebo pills during *Visit 5*. Assessments performed on the subjects during pre-intervention testing are summarized in Figure 2.

#### Randomization and blinding

Randomization is performed after subjects complete all screening measures, qualify for the study, and finish baseline testing. Although, we did not observe an obvious effect of subject (biological) sex or age on the change in FMD_BA_ with active treatment in our pilot study (Rossman et al., 2018): 1) the sample was likely too small (*n* = 20) to determine such effects; and 2) NIH requires that sex and other biological factors that may influence responsiveness be included in the analysis plan for purposes of rigor and reproducibility. Accordingly, block randomization has been employed based on biological sex (men and women) and age (60–70 years and >70 years). The study biostatistician developed the randomization tables and an unblinded research assistant, not involved with data collection or analysis, is responsible for individual subject group assignment. The CTRC nurses are blinded to the randomizations to maintain the integrity of the data and ensure accidental human error does not interfere with the randomization blinding. Investigative team members involved in the acquisition and analysis of outcome measurements are also blinded to group assignment.

#### Intervention administration and subject monitoring

Pill bottles containing either placebo or MitoQ are prepared and coded to keep subjects and investigators blinded to treatment condition. A member of the research team administers pills and provides dosing and timing instructions to subjects under the supervision of a CTRC nurse. Subjects are instructed to bring their pill bottles to each check-in visit (*Visits 6–8*; Figure 2) and post-intervention experimental visits (*Visits 9–11*; Figure 2) at which time a member of the research team counts the number of pills remaining in the container to assess adherence to the intervention.

During the intervention period, all subjects report to the CTRC once a month to meet with a CTRC nurse. The nurse asks subjects if they are experiencing any changes in their welfare and general health. The nurse also asks subjects if they are experiencing any side effects. The main side effect associated with MitoQ is gastrointestinal discomfort. As such, potential acute and chronic effects of MitoQ supplementation are assessed at all visits prior to and throughout the intervention using a symptom questionnaire and Visual Analog Scale (VAS). Subjects are instructed to contact the research coordinator or another member of the study team immediately if they develop any serious side effects or experience any change in their health. If subjects experience any serious unexpected side effects, they will be instructed to discontinue their respective intervention immediately. Transient minor side effects are closely monitored and continued participation in the study is determined by our physician of record.

#### Mid-intervention experimental assessment & protocol adherence

Following the completion of pre-intervention testing, the subjects return to the CTRC 2 weeks later for *Visit* 6 (Figure 2) to assess adherence to the protocol by returning previously received pill bottles (as discussed above); measure weight, resting blood pressure, and heart rate; complete a VAS; and receive a new allotment of pills for the next month.

One month later, subjects return to the CTRC for *Visit 7* for midpoint vascular testing. This testing includes measures assessing primary and secondary outcomes, including FMD_BA_, cfPWV, carotid compliance, intima media thickness, and β-stiffness index (Figure 2). *Visit 7* is 6 weeks from the start of the intervention and aligns with timing of measurements in our pilot trial with MitoQ supplementation (Rossman et al., 2018). To assess whether a longer duration of MitoQ supplementation (3 months) leads to a greater improvement in vascular function, midpoint vascular testing is performed and the change in endothelial function after 6 weeks will be compared with the change after 3 months of supplementation.

Subjects are asked to follow the same pre-intervention, pre-visit restrictions for *Visit 7*. In addition to midpoint vascular testing, subjects return pill bottles to assess adherence and receive an additional 1-month allotment of pills; bodyweight, blood pressure and resting heart rate are assessed; and subjects complete a symptom questionnaire and VAS.

One month later, subjects return to the CTRC for their final check-in visit, *Visit 8* (Figure 2). This visit is identical to *Visit 6*, except subjects receive enough pills to complete the remainder of the treatment.

#### Post-intervention experimental assessments

After completing 3 months of either MitoQ or placebo supplementation, participants return to the CU Boulder CTRC for post-intervention experimental assessments (*Visits 9–11*; Figure 2). Throughout post-intervention testing, subjects continue their respective intervention until the completion of post-testing. Subjects are allowed to take MitoQ or placebo the day before visits when outcomes are assessed (i.e., >10 h before testing) but asked to refrain from taking MitoQ the morning of testing to avoid acute effects on experimental testing. Subjects are asked to take their daily dose after their visit on testing days. *Visits 9–11* are identical to *Visits 3–5* in that they are conducted over 2 weeks and include identical outcome assessments. Assessments performed upon the subjects during post-intervention testing can be found in Figure 2.

#### Study withdrawal

Primary exit or stopping criteria for a given participant in the study includes completion of the study; withdrawal of informed consent (for any reason); an adverse event that, in the opinion of the physician of record, necessitates the participant stopping the study; the loss of ability to freely provide consent through imprisonment or involuntary incarceration for treatment of psychiatric or physical illness; significant non-compliance with the study protocols; or other significant (determined by the PI and physician of record) changes in medications, physical, or mental health.

#### Adverse events

Participants are instructed to inform members of the study team or CTRC staff of any side effects or adverse events. Anticipated mild and moderate adverse events are reported to the IRB on an annual basis. Serious adverse events will be reported to the CU Boulder IRB within 24 h of occurrence. An unanticipated adverse event which meets the CU Boulder IRB definition of an unanticipated problem (i.e., any unanticipated and undesirable effect arising from participation in research that results in an increased risk of harm or injury to a subject, or which suggests the possibility of increased risks to other subjects) will be reported to the CU Boulder IRB using the corresponding form within 5 days of occurrence.

### Statistical analyses

Sample size calculations and pre-determined statistical power for this randomized, double-blind, placebo-controlled, parallel group design clinical trial are based on the primary outcome (FMD_BA_) and the use of the two-sample *t*-test to compare the change in FMD_BA_ between groups following treatment. Preliminary data from our pilot study, which employed a randomized crossover design and was conducted in adults 60–80 years of age, was used to estimate the effect size to calculate sample size for a pre-specified statistical power (Rossman et al., 2018).

Based on the preliminary data from our crossover pilot trial, the increase in FMD_BA_ after treatment with MitoQ was 1.10%; a decrease of −0.38% was observed with placebo control yielding a difference between groups of 1.48% (Rossman et al., 2018). Of note, based on the relation between CVD risk and FMD in the peer-reviewed literature, a clinically meaningful difference in FMD_BA_ is 1.0% (Inaba et al., 2010). As such, the study is powered to achieve a difference in FMD_BA_ 50% greater than that considered to be a clinically meaningful change. The standard deviation of change was estimated as 2.50 and 2.10, respectively, under the two conditions, yielding a pooled standard deviation of 2.31. An effect size of 0.64 was calculated from the mean difference between groups and the pooled standard deviation (1.48/2.31 = 0.64), which was used to determine the required sample size.

At a statistical power of 85%, a two-sample *t*-test to compare the change in FMD_BA_ between groups, and a two-sided significance level of *α* = 0.05, 45 subjects per group (total of 90) is required. To account for a potential dropout of 20% (i.e., the rate observed in our pilot study) a total of 112 [112 (1.0–0.2) = 90] healthy men and women over 60 years of age will be enrolled and equally randomized to the two intervention groups with 56 subjects/group. While our sample size is based on an observed difference in FMD_BA_ that is greater than that considered clinically significant, our sample size has the added benefit of ensuring we are adequately powered to detect a clinically meaningful improvement in the primary outcome should the true effect size be lower than that observed in our pilot study.

Descriptive statistics will be provided for all baseline and outcome variables. The mean and standard error of the mean will be calculated for continuous variables, and proportion will be calculated for categorical or ordinal variables. Median and interquartile range (IQR) will be calculated if a continuous variable is skewed in distribution. The 95% confidence interval will be provided as appropriate. The effect of MitoQ treatment on a given outcome measure will be assessed by a linear regression model, such that post-intervention outcome measures will be regressed against both pre-intervention outcome measures and group randomization. Adjustment for covariates will also be explored.

Every effort will be made to include only participants that complete the trial in the final analysis. Since at least 45 subjects per group are expected to complete the study, a power of 85% or higher is anticipated if the real effect on the primary outcome (FMD_BA_) is similar to that of the pilot study (Rossman et al., 2018), i.e., 0.64. In an unlikely situation in which more than 20% of the enrolled participants drop out of the study, statistical analyses based on the intention-to-treat principle with multiple imputation will be performed. All conclusions will be made using a two-sided significance level of *α* = 0.05. Data transformation will be performed, if appropriate, before further analysis.

#### Data management

All subject identities and records remain strictly confidential. Only the principal investigator and research staff have access to data from this study. Individual subject data is coded with a unique identifier, is not associated with the subject’s name, and the codes are stored separately from any data. Physical data is stored in locking file cabinets in a secured-entry laboratory space. Electronic data is stored on a laboratory server, as well as REDCap, each of which require individual user sign-on and passwords. The names of the subjects will not be identified in any publication arising from this study. If individual participant data is presented in presentations or manuscripts, it will not be associated with any information that could be used to identify participants in the study.

#### Data safety monitoring

This research protocol is subject to oversight by an internal CTRC safety monitoring committee and an external safety officer, who is independent of the study team. The safety officer reviews safety data on at least an annual basis (using safety officer reports prepared by the research team) or as needed. Additionally, a physician investigator provides day-to-day medical oversight to ensure participant safety by serving as physician of record on this protocol. The study team is in regular contact with the physician of record and reviews enrollment and safety data with the safety officer at least twice per year.

## Expected results

### Primary hypothesis

1) Three months of chronic oral MitoQ supplementation will increase FMD_BA_, suggesting an improvement in endothelial function in healthy older adults.

### Secondary hypotheses

1) Improvements in endothelial function following chronic oral MitoQ supplementation in older adults will be mediated by reduced mtROS-related suppression of EDD. As such, the increase in FMD_BA_ following an acute 160 mg dose of MitoQ observed at baseline and under placebo conditions will be ameliorated following chronic oral MitoQ supplementation, suggesting MitoQ improves endothelial function by decreasing mtROS.
2) Chronic oral MitoQ supplementation will decrease cfPWV in healthy older adults exhibiting age-related aortic stiffening, suggesting a reduction in aortic stiffness.
3) Chronic oral MitoQ supplementation will increase carotid compliance and reduce β-stiffness index and intima media thickness, suggesting an improvement in large artery stiffness.

### Hypotheses for Other Outcomes

1) Chronic oral MitoQ supplementation will improve protein markers of oxidative stress and mitochondrial health in endothelial cells obtained via endovascular biopsy, as indicated by lower abundance of nitrotyrosine and Fis1.
2) NO bioavailability will increase and whole cell- and mitochondrial ROS will decrease in cultured endothelial cells exposed to serum obtained from subjects following MitoQ supplementation, demonstrating that alterations in circulating factors are, in part, responsible for improvements in endothelial cell function with chronic MitoQ supplementation.
3) Chronic oral MitoQ supplementation will improve circulating markers of oxidative stress, mitochondrial health, and inflammation as indicated by lower plasma oxLDL and myeloperoxidase; unchanged or increased total antioxidant status; lower levels of mtDNA, mitochondrial damage, and heteroplasmy of mtDNA; and lower IL-6 and C-reactive protein.

## Discussion

CVD is the leading cause of morbidity and mortality in the United States and aging is the strongest independent risk factor for the development of CVD (Lakatta, 2003; Lloyd-Jones, 2010; Benjamin et al., 2017). The increased risk of CVD with aging is largely mediated by the development of vascular dysfunction, particularly vascular endothelial dysfunction, characterized by reductions in EDD, and large elastic artery stiffness, each of which are independent predictors of CVD risk (Lakatta and Levy, 2003; Widlansky et al., 2003; Brandes et al., 2005; Yeboah et al., 2009; Mitchell et al., 2010; Seals et al., 2011; Ras et al., 2013). An important mechanism of age-related vascular dysfunction is excessive production of mtROS (James and Murphy, 2002; Lakatta and Levy, 2003; Fleenor et al., 2010; Seo et al., 2010; Seals et al., 2011; Fleenor, 2013; López-Armada et al., 2013), which reduces the bioavailability of NO leading to endothelial dysfunction and contributes to stiffening of large elastic arteries with aging (Rossman et al., 2020). Therefore, establishing the efficacy of interventions targeting mtROS, such as MitoQ, to improve vascular function with aging is of high biomedical importance.

We obtained initial evidence of efficacy of MitoQ for improving endothelial function and reducing arterial stiffness in a pilot clinical trial in older adults without clinical disease (Rossman et al., 2018). The main objective of this phase IIa clinical trial is to confirm and extend these promising pilot trial results by conducting a larger clinical trial of chronic oral MitoQ supplementation in older adults over a longer, more clinically relevant supplementation period (Whelton et al., 2017; Grundy et al., 2018) and to obtain insight into the mechanisms by which MitoQ improves vascular function in this group. To accomplish the latter, we employ innovative *in vivo* and *ex vivo* mechanistic assessments to determine the molecular mechanisms by which MitoQ favorably alters vascular function.

We do not foresee any significant problems executing this study as all the procedures are well established in our laboratory and we have a record of successful completion of clinical trials in older adults (Santos-Parker et al., 2017; Martens et al., 2018; Rossman et al., 2018; Martens et al., 2020; Craighead et al., 2021; Rossman et al., 2021). The conclusions of this study will further establish the efficacy of MitoQ for improving age-related vascular endothelial dysfunction and large elastic artery stiffness. If successful, the findings of the current trial may provide the basis for a larger, definitive phase III multi-site clinical trial to firmly establish MitoQ as a safe and effective clinical intervention to reduce CVD risk in older adults.

## Ethics and dissemination

### Research ethics approval

This study has been approved by the University of Colorado Boulder IRB (#20-0502).

### Protocol amendments

Amendments to improve the study protocol will be considered. Those deemed feasible and justified will be submitted to the IRB prior to implementation for approval.

### Confidentiality & access to data

As stated previously within the data management section, all subject identities and records remain strictly confidential. Only the principal investigator and research staff have access to data from this study. Individual subject data is coded with a unique identifier, is not associated with the subject’s name, and the codes are stored separately from any data. Because we need to link subject names and identification numbers to blood samples returned from the University of Colorado Anschutz Medical Campus, we keep a list of subject codes and names on a password-protected document on our laboratory server. Only authorized members of the study team have access to the password for this document. If a breach of this document should occur, the IRB will be notified immediately. This spreadsheet will be destroyed 1 year after the results of the study are published.

### Dissemination policy

This protocol is registered on ClinicalTrials.gov (Identifier: NCT04851288). Information regarding the trial record is updated at least once every 12 months through the ClinicalTrials.gov registry system. Results will also be reported on ClinicalTrials.gov within 1 year of study completion. Results from the study will be compiled and submitted for publication in a timely manner. Every effort will be made to ensure the data is published in an open access journal format to maximize impact and appropriate audience reach.
